# Free-grazing Ducks and Highly Pathogenic Avian Influenza, Thailand

**DOI:** 10.3201/eid1202.050640

**Published:** 2006-02

**Authors:** Marius Gilbert, Prasit Chaitaweesub, Tippawon Parakamawongsa, Sith Premashthira, Thanawat Tiensin, Wantanee Kalpravidh, Hans Wagner, Jan Slingenbergh

**Affiliations:** * Université Libre de Bruxelles, Brussels, Belgium;; † Department of Livestock Development, Bangkok, Thailand;; ‡ Utrecht University, Utrecht, the Netherlands;; § Food and Agriculture Organization, Bangkok, Thailand;; ¶ Food and Agriculture Organization, Rome, Italy

**Keywords:** Avian influenza, epidemiology, spatial analysis, Thailand, animal husbandry, research

## Abstract

Thailand has recently had 3 epidemic waves of highly pathogenic avian influenza (HPAI); virus was again detected in July 2005. Risk factors need to be identified to better understand disease ecology and assist HPAI surveillance and detection. This study analyzed the spatial distribution of HPAI outbreaks in relation to poultry, land use, and other anthropogenic variables from the start of the second epidemic wave (July 2004–May 2005). Results demonstrate a strong association between H5N1 virus in Thailand and abundance of free-grazing ducks and, to a lesser extent, native chickens, cocks, wetlands, and humans. Wetlands used for double-crop rice production, where free-grazing duck feed year round in rice paddies, appear to be a critical factor in HPAI persistence and spread. This finding could be important for other duck-producing regions in eastern and southeastern Asian countries affected by HPAI.

## Abstract 2

Free-grazing ducks in rice paddies are a critical factor in the spread and persistence of avian influenza.

Despite fears of an emerging influenza pandemic, human cases observed in Vietnam, Thailand, and Cambodia (*1*), and the severe socioeconomic losses in the poultry industry, the principal risk factors associated with the highly pathogenic avian influenza (HPAI) epidemic, which started in 2003 in eastern and southeastern Asia, are still poorly understood. Reports on the start of the epidemic in China indicated that a variety of H5N1 viruses circulated in domestic ducks in the coastal and southern parts of the country until the dominant Z strain emerged and caused a subcontinental-scale epidemic (*2**,**3*). Areas where both extensive and semi-intensive poultry production systems coexist were believed to be particularly at risk, while larger scale commercial and industrial poultry plants remained relatively unexposed (*4**,**5*). Recent studies found that ducks infected with H5N1 showed few clinical signs of disease (*3**,**6**,**7*) but were capable of shedding appreciable amounts of virus and may therefore form a potential reservoir or permanent source of infection. Trade and movements of live birds, including fighting cocks, and live-bird markets have also been identified as potential risk factors in the spread of HPAI caused by H5N1 (*5*).

Between January 2004 and early 2005, Thailand had 2 major HPAI epidemics (*8*). The first peaked at the end of January 2004 and a second, which may have started in July 2004, assumed epidemic proportions only after the end of September 2004 (Figure 1). On September 28, 2004, the Thai Government launched a nationwide survey (the x-ray survey) to produce a composite picture of HPAI situation in Thailand, reduce disease incidence, and when possible, halt virus circulation. This survey involved the participation of hundreds of thousands of inspectors searching door to door for evidence of HPAI. All sick and dead poultry in the villages suspected of HPAI infection were reported to local authorities.

**Figure 1:**
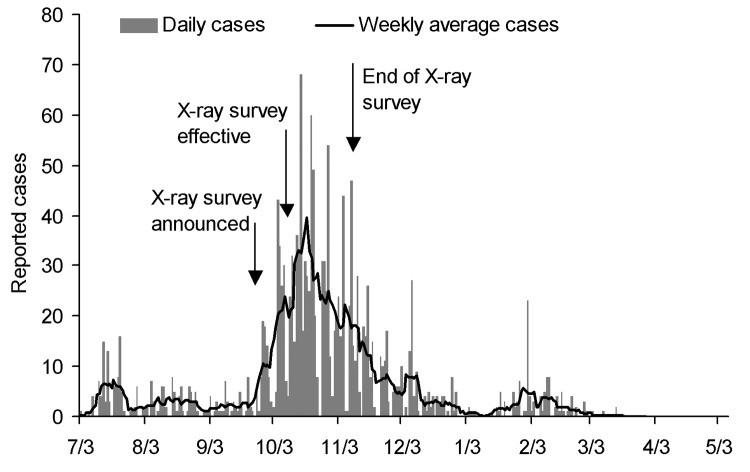
Number of daily highly pathogenic avian influenza outbreaks, Thailand, July 3, 2004–May 5, 2005. Shown are laboratory-confirmed H5N1 cases only, with the dates matching actual detection of clinical disease.

After initial training of inspectors, this operation was fully implemented in the second week of October 2004 until early November. This unprecedented increase in intensity of surveillance complicated the interpretation of records of disease outbreaks. The intensity of the second-wave epidemic was likely modulated by the x-ray survey because the increase in case detection activity contributed to a higher than usual number of reported HPAI outbreaks. Conversely, because of more intensive inspection and culling of infected birds, a more effective disruption of transmission cycles probably occurred, which contributed to a relatively strong decrease in incidence. However, the increase in reported cases just before the onset of the x-ray survey suggests that a serious outbreak was occurring. The weekly incidence of HPAI started to decrease at the end of October 2004, and the weekly number of disease outbreaks has continued to decrease progressively.

The aim of this study was to analyze the HPAI spatial distribution based on laboratory-confirmed H5N1 outbreaks recorded during the second epidemic. To identify the risk factors associated with HPAI, we applied autologistic multiple regression to relate HPAI to the geographic distribution of the main poultry species, relevant land-use features, and other environmental or anthropogenic variables.

## Materials and Methods

### Data

**Figure A1:**
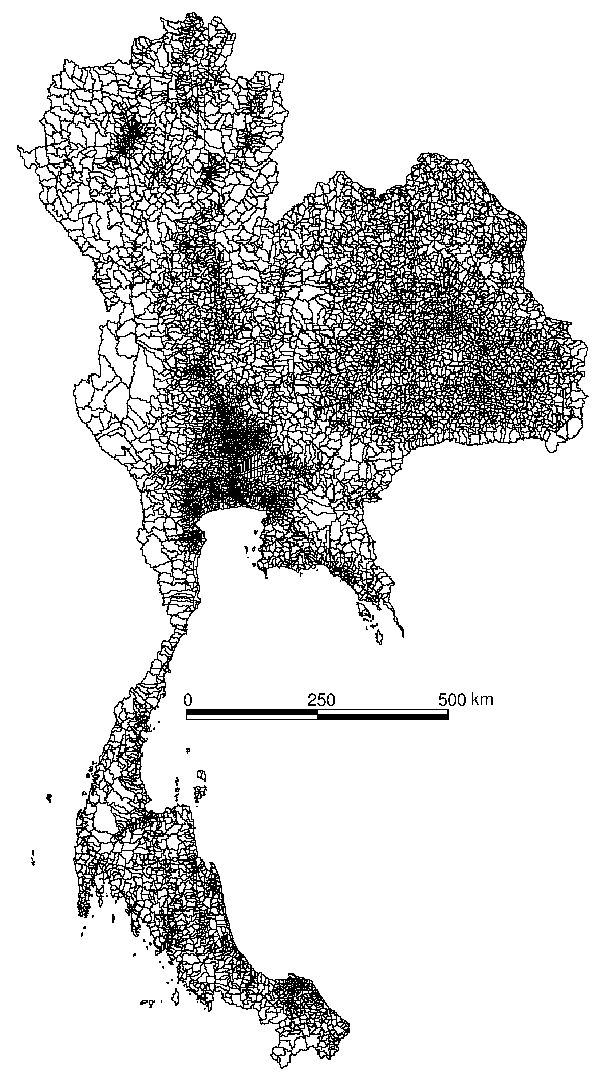
Distribution of Thailand subdistricts.

Data on HPAI outbreaks caused by H5N1 consisted of 1,716 laboratory-confirmed cases reported from July 3, 2004, to May 3, 2005, by the Department of Livestock Development, Ministry of Agriculture and Cooperatives, Bangkok, Thailand. These data were pooled for the entire time series and converted into presence or absence of HPAI within each of the 8,089 subdistricts of Thailand (Figure A1) for analysis at the national level and within each of the 913 villages in Suphanburi Province for analysis at the local level. Poultry census data were collected simultaneously in the x-ray survey from all Thai villages countrywide during October to November 2004. Poultry data comprised bird numbers and categories by subdistrict for analysis at the national level or by village for analysis in Suphanburi Province.

Poultry categories considered in the analysis were farm chickens (including broilers and layer hens), native chickens, farm ducks (including meat and layer ducks), free-grazing ducks (domestic ducks raised in the open in flocks of >1,000 birds for egg production and, to a lesser extent, for meat; see Discussion for a more detailed description of this type of husbandry), cocks, and other poultry. Native chickens and free-grazing ducks form separate categories as because both groups are raised in the open and are more exposed to prevailing pathogens. In contrast, variable levels of biosecurity measures may apply to chickens and ducks that are raised in farms.

In addition to poultry data (Table 1), we obtained relevant variables describing road network, land use, and physical environment (*9*). These variables were used to analyze 1) possible disease introduction and propagation through waterfowl's frequenting rice paddy fields and wetlands (thus the choice of variables relating to the rice fields, wetlands, and topography), and 2) the role of human activities in the spread of disease associated with live-bird trade and traffic (thus the choice of variables on human population and roads).

**Table 1:** Variables used in the analysis of highly pathogenic avian influenza distribution in Thailand (subdistrict level) and Suphanburi Province (village level)

Category	Description	Abbreviation
Poultry*	No. broilers and layer hens	BrLaCh
	No. native chickens	NaCh
	No. meat and layer ducks	MeLaDu
	No. free-grazing ducks	FgDu
	No. cocks	Co
	No. other poultry	Ot
Land use/water†	Proportion of land occupied by wetland in the subdistrict, and in a 1-, 2-, 5-, and 10-km radius neighborhood	Pwet, Pwet1K, Pwet2K, Pwet5K, Pwet10K
	Proportion of land occupied by rice fields in the subdistrict, and in a 1-, 2-, 5-, and 10- km radius neighborhood	Price, Price1K, Price2K, Price5K, Price10K
	River/stream density (per km^2^) in the subdistrict and in a 1-, 2-, 5-, and 10-km radius neighborhood	Sd, Sd1K, Sd2K, Sd5K, Sd10K
People/roads‡	No. humans	Hpop
	No. roads	Nroads
Topographic§	Elevation, m	Alt
	Slope, degrees	Slp

*Census in the subdistrict or village. †Estimate in the subdistrict was not used for the village-level analysis; distance-based variables between the 1- and 10-km neighborhood were estimated around the village point or subdistrict centroid. ‡No. roads in the subdistrict or connected to the village. §These variables were averaged in the subdistrict polygon for subdistrict level analysis or extracted at the point of the village for village-level analysis.

### Statistical Analysis

Preliminary analysis on HPAI distribution in Thailand indicated that Suphanburi Province accounted for nearly 50% of all outbreaks in ducks (outbreaks in ducks refer to outbreaks reported in all type of domestic ducks). This province had the highest cumulative number of outbreaks and a large population of free-grazing ducks. We thus decided to conduct a follow-up analysis of HPAI distribution in Suphanburi Province using village-level data on HPAI presence or absence. Therefore, this 2-scale analysis, in addition to considering an identical analytical approach for 2 different levels of resolution, also compares results obtained at the national level, including areas where HPAI outbreaks were never reported, with those obtained of the epicenter of HPAI in Thailand.

The association between HPAI occurrence, either at the subdistrict or village level, and the poultry and environmental variables was explored by using stepwise multiple logistic regressions. Linear model statistics are affected by spatial autocorrelation in response and predictor variables, i.e., the tendency for the value of neighboring points to be more similar than those from distant points. This tendency, known as spatial autocorrelation, contradicts the assumption of independence among samples replicated through space (*10*). We accounted for spatial autocorrelation in the general model by applying an autologistic approach (*11**,**12*), in which an autologistic term was added as a covariate to the logistic model (the autologistic term averages the probability of HPAI presence among a set of neighbors, defined by the limit of autocorrelation and weighted by the inverse of the Euclidean distance). The extent of the autocorrelation of the response variable was obtained from the spatial correlogram ρ(h) (*13*) of HPAI presence or absence. The inverted correlogram 1 – ρ(h) was modeled by using a spherical model (*14*), and the parameters for the model (termed nugget, scale, and range, respectively) were obtained by using nonlinear regression with bootstrapped estimates of the standard errors (SPSS version 12.0; SPSS Inc., Chicago, IL, USA). The autoregressive term was built by using a neighborhood determined by the range of the spatial correlogram model and was estimated as the average number of HPAI instances in this neighborhood weighted by the inverse distance. The autoregressive term was then added to each tested model.

A first ranking and selection of variables consisted of testing the HPAI status separately against each variable (with the autoregressive term included), and variables yielding nonsignificant changes in log-likelihood were excluded. Next, a stepwise multiple logistic regression with forward entry mode was carried out by using the subset of variables and entering the variable accounting for the highest change in the model log-likelihood. This procedure was repeated until no additional significant variable could be added (likelihood ratio test; decision rule: p<0.01 for entry, p>0.05 for removal). The regression with the subset of variables was also run in backward mode, and the most parsimonious model included the variables found significant, and with the same sign, using the 2 approaches.

The performance of the models was assessed by determining the area under the curve (AUC) of the receiver operating characteristics plots. AUC is a quantitative measure of the overall fit of the model that varies from 0.5 (chance event) to 1.0 (perfect fit) (*15*). This measure is independent of the threshold value (*16*) and has the advantage of being independent of presence rarity, which is not the case with Cohen's kappa index.

## Results

**Figure 2:**
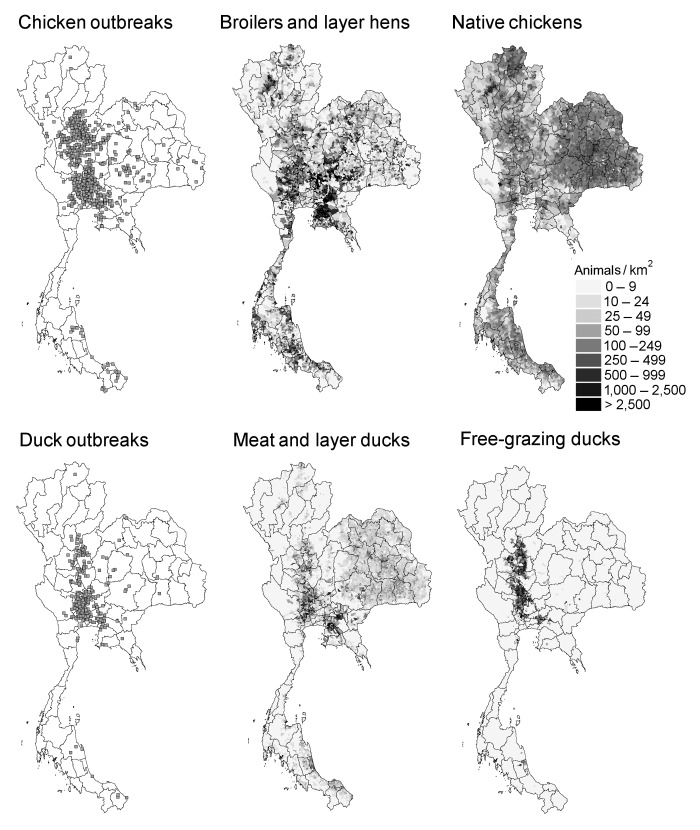
Distribution of highly pathogenic avian influenza (HPAI) outbreaks in chickens and ducks, Thailand, July 3, 2004–May 5, 2005, and respective distribution of broilers and layers hens, native chicken, meat and layer ducks, and free-grazing duck populations, highlighting the correlation between HPAI outbreak distribution and free-grazing duck populations. The divisions are Thailand provinces.

Most H5N1 outbreaks in poultry in Thailand were recorded in chickens (*8*). However, the distribution of these clinical outbreaks in chickens did not match the distribution of native, backyard chickens (Figure 2). Instead, the national distribution of HPAI outbreaks shows the strongest association with the distribution of free-grazing ducks (Figure 2). This result is quantified in Tables 2 and 3, which shows the number of free-grazing ducks as the most important risk factor associated with HPAI presence (as quantified by the Wald statistic). HPAI presence is also, but to a lesser extent, associated with number of native chickens, land elevation, number of cocks, and size of the human population. Elevation is the only variable that shows a negative association with HPAI presence, which shows that most outbreaks occurred in the lower plains.

**Table 2:** Parameters of autologistic regression models of highly pathogenic avian influenza presence or absence as a function of selected poultry and other environmental variables

Model	Variable*	Parameter	SE	Wald statistic	p value
Thailand, all outbreaks					
	Art	37.03	1.69	482.5	<0.001
	FgDu	2.47 × 10^-5^	3.67 × 10^-6^	45.3	<0.001
	NaCh	1.63 × 10^-5^	4.18 × 10^-6^	15.1	<0.001
	Alt	–3.60 × 10^-3^	1.00× 10^-3^	12.9	<0.001
	Co	1.12 × 10^-4^	3.21 × 10^-5^	12.1	<0.001
	Hpop	1.03 × 10^-5^	3.10 × 10^-6^	10.9	<0.001
	Alt2	2.40 × 10^-6^	1.01 × 10^-6^	5.7	0.017
	Intercept	–2.93	0.16	317.9	<0.001
Thailand, outbreaks in chicken					
	Art	44.49	2.33	363.2	<0.001
	FgDu	1.76 × 10^-5^	3.25 × 10^-6^	29.5	<0.001
	NaCh	1.62 × 10^-5^	4.23 × 10^-6^	14.7	<0.001
	Alt	–3.91 × 10^-3^	1.03 × 10^-3^	14.6	<0.001
	Alt2	2.73 × 10^-6^	9.96 × 10^-7^	7.5	0.006
	Co	7.55 × 10^-5^	2.98 × 10^-5^	6.4	0.011
	Hpop	7.85 ×10^-6^	3.50 × 10^-6^	5.0	0.025
	Intercept	–2.92	0.17	302.9	<0.001
Thailand, outbreaks in ducks					
	Art	41.60	2.87	209.9	<0.001
	FgDu	2.94 × 10^-5^	3.65 × 10^-6^	64.9	<0.001
	Alt	–1.15 × 10^-2^	2.16 × 10^-3^	28.4	<0.001
	Alt2	7.63 × 10^-6^	1.83 × 10^-6^	17.4	<0.001
	Price10K	9.24 × 10^-1^	2.39 × 10^-1^	14.9	<0.001
	Co	4.78 × 10^-5^	1.66 × 10^-5^	8.2	0.004
	Hpop	1.22 × 10^-5^	4.65 × 10^-6^	6.9	0.009
	MeLaDu	5.96 × 10^-6^	2.66 × 10^-6^	5.0	0.025
	Intercept	–3.33	0.31	112.2	<0.001
Suphanburi Province, all outbreaks					
	Price5K	3.70	0.621	35.6	<0.001
	FgDu	9.46 × 10^-5^	2.03 × 10^-5^	21.7	<0.001
	MeLaDu	7.47 × 10^-5^	2.52 × 10^-5^	8.8	0.003
	NaCh	2.09 × 10^-4^	8.27 × 10^-5^	6.4	0.012
	Intercept	–4.89	0.511	91.6	<0.001
Suphanburi Province, outbreaks in chickens					
	Price5K	4.64	1.135	16.7	<0.001
	NaCh	3.01 × 10^-4^	1.00 × 10^-4^	9.0	0.003
	MeLaDu	7.00 × 10^-5^	2.60 × 10^-5^	7.2	0.007
	FgDu	4.54 × 10^-5^	1.81 × 10^-5^	6.3	0.012
	Intercept	–6.74	0.960	49.3	<0.001
Suphanburi Province, outbreaks in ducks					
	Price5K	3.76	0.742	25.7	<0.001
	FgDu	8.67 × 10^-5^	1.87 × 10^-5^	21.4	<0.001
	MeLaDu	7.16 × 10^-5^	2.39 × 10^-5^	9.0	0.003
	Intercept	–5.16	0.604	73.1	<0.001

*SE, standard error; Art, autoregressive term. For descriptions and abbreviations of other variables, see Table 1.

**Table 3:** Results of autologistic regression models of H5N1 highly pathogenic avian influenza as a function of variables shown in Table 2

Model	–2 log likelihood	χ^2^	p value	AUC*
All Thailand, all outbreaks	3,812.8	1,294.3	<0.001	0.854 ± 0.014
All Thailand, outbreaks in chickens	3,455.9	812.9	<0.001	0.828 ± 0.018
All Thailand, outbreaks in ducks	1,634.7	781.7	<0.001	0.894 ± 0.021
Suphanburi Province, all outbreaks	691.4	135.8	<0.001	0.783 ± 0.039
Suphanburi Province, outbreaks in chickens	374	63.05	<0.001	0.794 ± 0.061
Suphanburi Province, outbreaks in ducks	585.8	106.8	<0.001	0.767 ± 0.045

*Area under the curve of the receiver-operating characteristics plot.

When results are further analyzed in terms of chicken and duck HPAI outbreaks separately, for outbreaks in ducks, the association with native chickens is no longer present, while a positive association is observed with the proportion of rice paddy fields in the 10-km range neighborhood and the number of farms and free-grazing ducks. When the analysis was carried for Suphanburi Province at the village level, results were consistent with those obtained in the national-level analysis. This analysis included the association with the number of ducks (free-grazing ducks and meat and layer ducks), proportion of rice paddy fields in the 5-km neighborhood, and the number of chicken, both for all outbreaks and for chicken outbreaks only.

**Figure A2:**
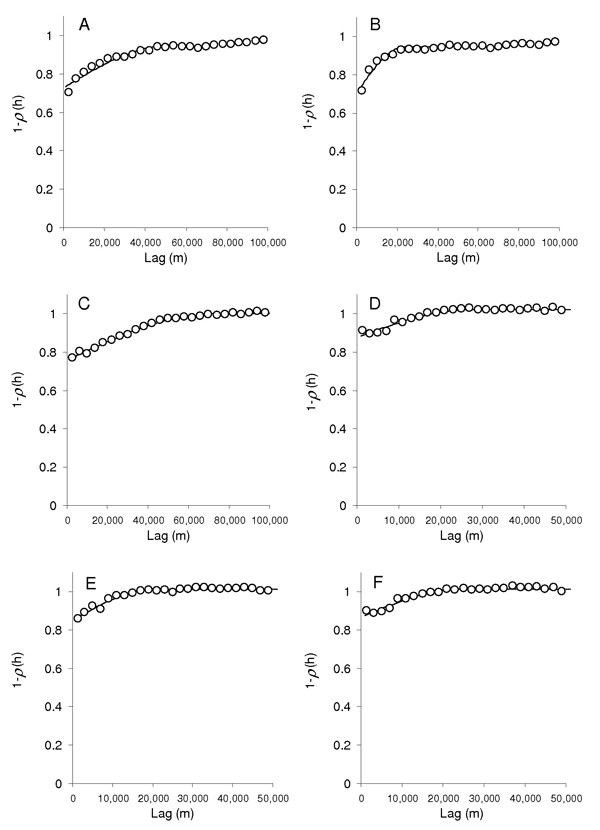
nverted spatial correlograms of highly pathogenic avian influenza presence or absence in Thailand. A) all outbreaks; B) outbreaks in chickens; C), outbreaks in ducks; D) all outbreaks in Suphanburi Province; E) outbreaks in chickens in Suphanburi Province; F) outbreaks in Suphanburi Province in ducks.

These results, particularly the association of HPAI with free-grazing ducks, are maintained when the analysis was stratified for 3 study periods: the period before the start of the x-ray survey (July 3–September 28, 2004), during the x-ray survey period (September 28–November 10, 2004), and beyond (November 10, 2004–May 5, 2005). The spatial structure of HPAI presence or absence as quantified by their spatial correlograms (Figure A2 and Table A1). This was characterized by a relatively weak spatial dependence with all scale parameters estimated as <0.25 (scale parameter measures the intensity of spatial autocorrelation and ranges between 0 and 1) and an estimated range between 20 km and 72 km (range parameter measures the geographic extent of the spatial autocorrelation).

## Discussion

Although most HPAI outbreaks during the second epidemic in Thailand occurred in chickens, the spatial distribution of these outbreaks does not correspond to areas with high densities of chickens. For example, northeastern Thailand has many native chickens that are not protected by biosecurity measures. However, apart from incidental HPAI outbreaks, this disease never showed a marked increase in this area (Figure 2). Instead, the distribution pattern suggests an important role of free-grazing ducks in rice paddies as in the central plains of Thailand. The variable genetic susceptibility of different poultry species, breeds, or races to HPAI may have created a bias in the recorded results, given that clinical detection of HPAI was the measure of HPAI presence.

**Figure 3:**
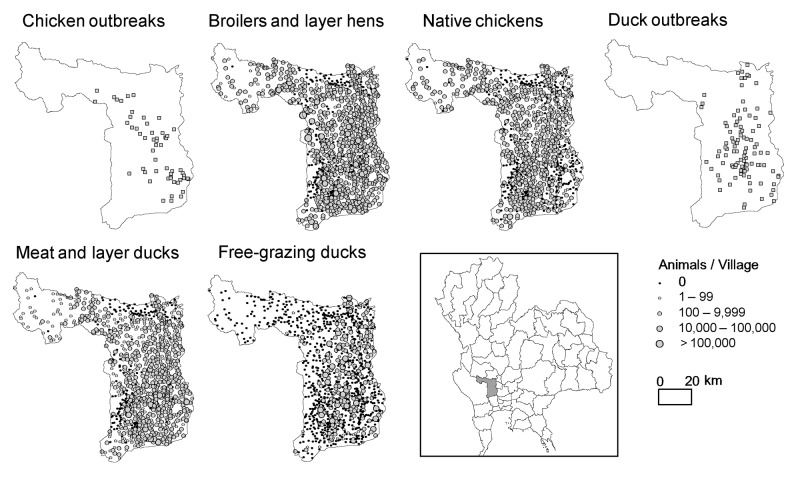
Distribution of highly pathogenic avian influenza (HPAI) outbreaks in chickens and ducks, Thailand, July 3, 2004–May 5, 2005, and respective distribution of broilers and layers hens, native chicken, meat and layer ducks, and free-grazing duck populations.

Although subsequent H5N1 verification was carried out for all reported outbreaks, virus circulation in native chickens may have remained unnoticed because disease presence was not prominent. Furthermore, the reduced susceptibility of ducks has likely contributed to underreporting of HPAI virus because ducks may carry virus but have no signs of disease (*3**,**6**,**17*). Nevertheless, the results substantiate the claim that the geographic pattern of HPAI outbreaks in Thailand is not primarily driven by long-distance transmission between chicken productions units or villages, which would have resulted in more outbreaks in areas with high densities of chickens. In the national level analysis, free-grazing ducks constitute the most important poultry-associated variable associated with HPAI in either ducks or chicken (Table 2). The significant but weaker association found for native chickens, both in the analysis of all HPAI reports and of HPAI reports in only chickens, may reflect infections in areas with a higher abundance of the host. This association is confirmed because this variable was replaced by duck numbers in the analysis of HPAI presence in ducks. The pattern that emerges is that free-grazing ducks form a HPAI risk factor both in chicken and ducks, which suggests that they may form a reservoir of HPAI virus. Conversely, chicken and duck numbers are associated with the probability of an outbreak in each respective category, i.e., they are related to the occurrence of infections. The robust association between HPAI and free-grazing ducks at the national level (Figure 2) corroborates the results obtained for Suphanburi Province (Figure 3). Ducks are the type of poultry most strongly associated with HPAI presence in villages.

Traditional free-grazing duck husbandry in Thailand is characterized by the practice of frequent rotation of duck flocks in rice paddy fields after the harvest, in which they are moved from 1 field to another every 2 days to feed on leftover rice grains, insects, and snails. Duck husbandry involves frequent field movements of flocks that are brought together in shelters often located within villages; with marketing of live birds and eggs extending beyond villages, apparently healthy ducks may play an important role in virus transmission, which explains the observed spatial pattern of HPAI. Infectious poultry or livestock diseases can be transmitted either locally through contagion between adjacent production units; by direct contact; by wind, insects, or rodents; or over a long distance by movements of animals, persons, or infected material (*18*). Local spread typically results in a strong spatial clustering of cases, whereas long-distance spread produces a distance-independent distribution of cases. The weak spatial autocorrelation in HPAI presence or absence, in particular in Suphanburi Province, indicates a weak clustering of HPAI. This finding suggests a relatively important contribution of long-distance movements of animals and infected materials.

**Figure 4:**
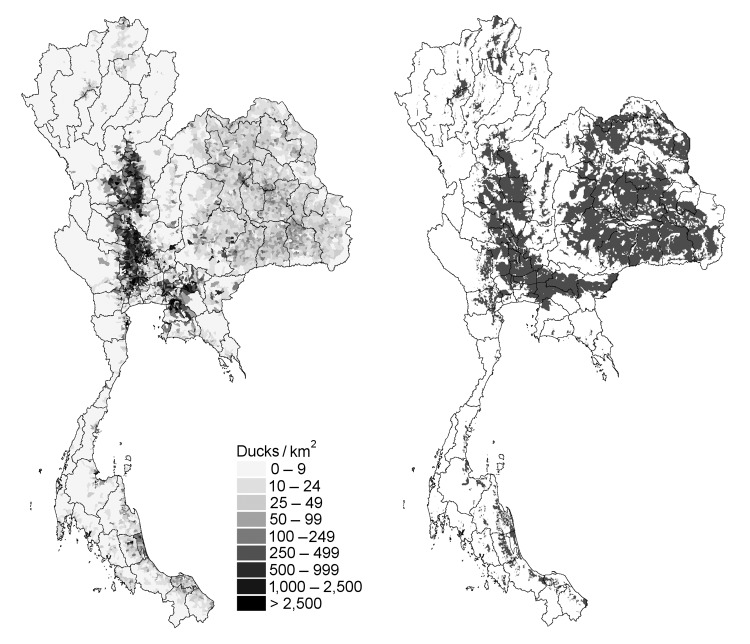
Distribution of A) duck and B) rice production areas in Thailand.

The duck production cycle is closely connected with rice crops because rice provides duck feed. Figure 4 shows the distribution of duck and rice paddy fields. Most rice fields in eastern Thailand produce 1 crop per year, but areas in the central plains (Figure 4B) produce 2 or even 3 crops per year. Single-crop areas are associated with duck farming, but fewer ducks are present because of the shorter period of rice harvest. In contrast, in double-crop areas, rice paddy fields are available year round after harvest. This availability sustains the low-input, low-output, free-grazing duck farming system and represents a large proportion of total ducks. As shown in Figure 4, there is a good correlation between the distribution of ducks and rice paddy fields and a strong correlation between free-grazing duck areas and double rice–crop areas in the central plains.

The 2-crop rice production system in the central plains is facilitated by local hydrology because irrigation systems provide enough water and wetland to produce a second crop outside the monsoon period. These wetlands and feed in the paddy fields are also attractive to migratory waterfowl and create a meeting point for wild and domestic aquatic bird species. The coexistence of free-grazing ducks and waterfowl during a defined period of the year (mainly November to February) may have provided an entry point or an index case for HPAI in poultry population in Thailand. The positive association between HPAI in villages in Suphanburi Province and the proportion of rice fields around the village, and the negative association with elevation (reflecting that HPAI was more frequently found in lower wetlands) suggest that wetland-rice-duck systems increase the risk for HPAI outbreaks, even after the effect of free-grazing ducks has been considered.

The strong association between ducks and rice crops facilitates application of remote sensing to identify rice-crop areas and patterns that may sustain forms of duck husbandry prone to HPAI outbreaks. All currently affected countries are known for their rice and duck production. For example, similar associations between rice and duck farming occur in Vietnam, where HPAI-affected areas coincide with river delta areas with year-round rice production. With duck populations remaining relatively healthy while excreting sufficient amount of virus to sustain transmission (*6*), wetlands with duck-production areas may act as a reservoir from which the virus can spread to distant aquatic duck farms and terrestrial chicken farms.

Other factors have been proposed as potential pathways for the spread of HPAI. These include migratory birds and introductory spread of virus from disease-endemic sources in China (*19*), trade of live animals and animal products (e.g., restocking, movements to slaughterhouses) within and away from infected areas, and movement of fighting cocks. Insufficient information exists to discern the possible role of migratory birds. However, several hypotheses have been proposed regarding their role and contribution to observed patterns of HPAI outbreaks. For example, a plausible scenario is that migratory birds initially spread H5N1 virus genotype Z virus over wide areas, but HPAI increased only after transmission to free-grazing ducks through water contamination, resulting in local amplification, persistence, and secondary spread to terrestrial poultry.

We found significant associations at the national level between HPAI and the overall number of cocks used in cock fights. The results at the national level also suggest that human activities may have played a role through a higher risk for transmission in more densely populated areas where poultry-related trade and traffic are more intensive. However, since these results were less strongly associated with HPAI and were not important at the village level, follow-up and local analysis of disease hotspots are needed to confirm that these 2 factors substantially contributed to transmission of HPAI, mainly within terrestrial poultry.

Options are available to veterinary authorities to further contain HPAI persistence in the central plains, address frequent movement of duck flocks in rice paddy fields, especially at the time of wild-bird migration, and actively encourage duck production in farms with adequate biosecurity. In 2005, a number of new control measures were introduced to enhance HPAI prevention, persistence, and spread nationwide. Some of these control measures specifically target free-grazing duck husbandry. These measures included registration and surveillance of all flocks (culling infected animals and compensating their owners), premovement testing, and incentives for improving biosecurity and shifting from free-grazing duck husbandry to farm production systems. These measures were effective in reducing the number of HPAI outbreaks in 2005. A total of 1,064 outbreaks were reported from July 3 to October 31, 2004 (second epidemic wave), but only 64 outbreaks were recorded during the same period in 2005. These results show that HPAI was still in Thailand in late 2005. Whether these outbreaks result from year-round persistence of HPAI within Thailand or from new introductions from external sources remains to be established. The reduced number of outbreaks suggests an overall reduction in circulation of the virus in free-grazing ducks and terrestrial poultry and a reduced risk for spread to birds or mammals.

In conclusion, our results highlight that free-grazing ducks were a critical factor in HPAI persistence and spread in Thailand during the second HPAI epidemic in 2004 at a time when there was little regulation concerning their movements and potential transmission to terrestrial poultry. This finding is of particular importance to duck-producing regions in other countries affected by HPAI.

## Appendix

**Table A1:** Estimated values ± standard error for the nugget, scale, and range of the best-fit spherical models of the spatial correlograms of highly pathogenic avian influenza presence or absence

Model	Nugget	Scale	Range (km)	r^2^
Thailand, all outbreaks	0.727 ± 0.011	0.222 ± 0.012	52.41 ± 3.66	0.946
Thailand, outbreaks in chickens	0.703 ± 0.017	0.243 ± 0.017	23.88 ± 2.02	0.925
Thailand, outbreaks in ducks	0.703 ± 0.004	0.243 ± 0.005	72.41 ± 2.11	0.990
Suphanburi Province, all outbreaks	0.875 ± 0.007	0.146 ± 0.007	27.35 ± 1.90	0.946
Suphanburi Province, outbreaks in chickens	0.848 ± 0.008	0.163 ± 0.008	20.20 ± 1.29	0.954
Suphanburi Province, outbreaks in ducks	0.866 ± 0.007	0.148 ± 0.007	25.05 ± 1.60	0.953
